# Autoimmune neuropsychiatric sequelae of group A streptococcal infections in Aboriginal and Torres Strait Islander people: an overlooked burden?

**DOI:** 10.1177/10398562251351462

**Published:** 2025-06-17

**Authors:** Sarangan Ketheesan, Vicki Wade, Te-Yu Hung, Kathryn V Roberts

**Affiliations:** Faculty of Medicine, 420004 The University of Queensland, Herston, QLD, Australia; National Centre for Youth Substance Use Research, The University of Queensland, Herston, QLD, Australia; Metro North Mental Health Service, Queensland Health, Brisbane, QLD, Australia; 10095 Menzies School of Health Research, Charles Darwin University, Darwin, NT, Australia; 10095 Menzies School of Health Research, Charles Darwin University, Darwin, NT, Australia; Department of Paediatrics, 71507 Royal Darwin Hospital, Darwin, NT, Australia

**Keywords:** Sydenham’s chorea, PANDAS, autoimmune, streptococcal infection, Aboriginal, Torres Strait Islander

## Abstract

**Objective:**

We highlight two autoimmune neuropsychiatric sequelae of group A streptococcal (GAS) infection that may cause disproportionate levels of psychiatric morbidity in Aboriginal and Torres Strait Islander people: Sydenham’s Chorea (SC) and Paediatric Autoimmune Neuropsychiatric Disorders Associated with Streptococcal Infections (PANDAS). GAS infections, along with better characterised immune-mediated sequelae such as acute rheumatic fever and rheumatic heart disease, are highly prevalent in Aboriginal and Torres Strait Islander people, particularly in rural and remote communities, yet there is minimal information in the literature about the neuropsychiatric sequelae that might be expected. We suspect that there is an under-recognised burden of disease in these groups and seek to understand reasons for this.

**Conclusions:**

A multi-faceted, culturally attuned approach to better characterising and detecting the burden of these two conditions in Aboriginal and Torres Strait Islander people is required. Furthermore, larger scale clinical trials examining the efficacy of proposed treatments for SC and PANDAS are required to inform clinical guidelines.

Despite efforts aimed at ‘Closing the Gap’, disparities in health outcomes in Aboriginal and Torres Strait Islander people persist.^1^ The infectious disease burden in these groups, especially for those dwelling in rural and remote areas, is particularly pronounced.^1^ Factors that underpin the burden of infectious disease – including group A streptococcal (GAS) infections – in these groups include poor health and sanitation infrastructure, over-crowding and lack of access to primary healthcare facilities.^2^

The incidence of GAS infections, responsible for conditions such as impetigo and pharyngitis, is significantly greater in Aboriginal and Torres Strait Islander people than in non-Indigenous Australians.^3^ Well-characterised immune-mediated sequelae of GAS infections such as acute post-streptococcal glomerulonephritis (APSGN), and acute rheumatic fever (ARF) are known to disproportionately affect these groups.^2^ The age standardised first-ever incidence rate of ARF, for example, is known to be 99 times greater in Aboriginal and Torres Strait Islander people than in non-Indigenous Australians.^4^ As such, we expect that neuropsychiatric sequelae of GAS infections in these groups are also likely to occur at higher rates than in non-Indigenous Australians. We posit that is particularly the case with Sydenham’s Chorea (SC), which is a diagnostic feature of ARF.

This perspective piece aims to highlight autoimmune-mediated post-streptococcal sequelae as an under-reported source of psychiatric morbidity that is likely to disproportionally affect Aboriginal and Torres Strait Islander people. We specifically examine SC and Paediatric Autoimmune Neuropsychiatric Disorders Associated with Streptococcal Infections (PANDAS). We also discuss social and cultural factors that may affect accurate reporting, detection and timely management of these conditions, and propose ways forward in addressing the burden of SC and PANDAS in Aboriginal and Torres Strait Islander populations.

## Sydenham’s chorea

SC is a major diagnostic criterion for ARF.^5^ It has a preponderance in females and a peak incidence in children aged 5-15 years.^6^ Although its most obvious feature is the movement disorder (chorea), psychiatric features of SC can mimic obsessive-compulsive disorder (OCD), depression and anxiety, attention-deficit hyperactivity disorder (ADHD) and even psychosis.^7^ While the course of the chorea itself is usually self-limited to a number of weeks (although can recur),^8^ longer-lasting deficits in cognition and executive function are increasingly recognised.^9^ A recent retrospective case series of 110 children with SC from the Northern Territory (109 of whom were Aboriginal) described irritability and mood lability as the most commonly reported neuropsychiatric features. Although only 34% of the cohort had specific enquiry about neuropsychiatric symptoms, symptoms were reported by 89% of the cohort.^5^ Larger prospective cohort studies examining the neuropsychiatric manifestations of SC specifically in Aboriginal and Torres Strait Islander people are lacking.

The mechanisms that mediate the neuropsychiatric symptoms of SC are yet to be fully characterised. Current hypotheses postulate that antibodies triggered by GAS infections (in particular antibodies against a specific epitope known as the M protein) cross-react with specific proteins in the basal ganglia. These basal ganglia proteins include neuronal ganglioside, lysoganglioside, N-accetyl-beta-glucosamine, dopamine 1 (D1) and dopamine 2 (D2) receptors. Immune-mediated disruption of dopaminergic transmission in the basal ganglia, caused by this cross-reactivity, is thought to be a potential driver of the neurological and psychiatric symptoms of SC.^7^

The management of all forms of definite ARF and RHD includes monthly, long-acting penicillin injections (‘secondary prophylaxis’) for a minimum of 10 years to reduce GAS infection and prevent ARF recurrences. This is a challenging treatment and adherence rates are often low.^10^ Specific treatments for SC include those targeting symptoms and those targeting the underlying immune process. Symptomatic treatments include the use of anticonvulsants, neuroleptics and tetrabenazine.^5,7,11^ Immunomodulatory treatments for SC include corticosteroids and intravenous immunoglobulin (IVIg) and aim to suppress the immune processes that mediate the symptomatology of SC.^12^ It is important to highlight that there are no published large scale trials available to guide clinical decision-making in the management of SC; guidelines are consensus-based, and there is significant variation in clinical practice.^5,7,8^ Despite the lack of robust clinical trials, a recent comprehensive meta-analysis highlighted the utility of corticosteroids, sodium valproate and antibiotics in the treatment of SC.^13^

## PANDAS

PANDAS is classified under the category of Paediatric Acute-onset Neuropsychiatric Syndromes (PANS).^14^ The diagnosis of PANDAS is contingent on five clinical criteria: the presence of OCD or tic disorder, symptom onset prior to puberty, an acute onset and episodic course, temporal association with GAS infection and association with neurological abnormalities.^14^ PANDAS has yet to be studied in Aboriginal and Torres Strait Islander people and there is a lack of epidemiological data on its prevalence globally, although it is thought to have a preponderance in males.^15^ Aside from OCD, PANDAS is thought to be associated with a range of neuropsychiatric symptoms which have a considerable degree of overlap with SC including: anxiety, aggression, hyperactivity, perceptual disturbances and disordered eating.^7^

Current understandings of the pathophysiology of PANDAS are limited, with mechanisms thought to be similar to those that mediate SC. Antibodies produced following a GAS infection are thought to target proteins in the basal ganglia including lysoganglioside, tubulin and D1 and D2 receptors, via a mechanism known as molecular mimicry.^15^ More recently, antibody-mediated inhibition of striatal cholinergic interneurons is thought to be implicated in the pathophysiology of PANDAS.^16^

Clinical management of PANDAS targets three domains, the first of which involves the treatment and prevention of recurrent GAS infections through means such as antibiotics and even prophylactic tonsillectomy and adenoidectomy.^17^ Immunomodulatory therapies (including corticosteroids, IVIg and plasma exchange) are also employed, along with psychiatric and behavioural treatments (such as cognitive behavioural therapy and selective serotonin reuptake inhibitors for OCD).^15,18^ Management is directed by consensus guidelines, in the absence of high-level evidence. It is pertinent to note that the PANDAS concept has been subject to a high degree of deliberation, conjecture and controversy since its inception over two decades ago.^15^ This is in part due to the inability to reliably demonstrate temporal and causal relationships between GAS infection and symptom onset, as well as the absence of randomised control trials to demonstrate treatment efficacy.^15^

## Cultural considerations

It is important to acknowledge that a cultural lens should be applied to all psychiatric conditions, regardless of how biologically driven they may appear. This is because cultural factors influence the diagnosis, formulation, treatment response and prognosis of all psychiatric disorders.^19^ Vance et al recently illustrated how culturally informed assessments can lead to meaningful differences in the presentation of psychiatric symptoms among Aboriginal and Torres Strait Islander young people.^20^ Despite equivalent clinical impairment to a matched non-Indigenous group, the nature and context of symptoms in the Aboriginal and Torres Strait Islander group were interpreted differently due to varying cultural frameworks.^20^ This insight is relevant to neuropsychiatric syndromes such as SC and PANDAS, where cultural factors can influence the presentation and diagnosis of such conditions and may also determine the acceptability and success of treatment approaches.

Cultural factors may also lead to under-detection of these conditions. Intergenerational trauma leading to mistrust of health services and the stigma that is often attributed to psychiatric symptoms are two pertinent cultural factors that may lead to under-reporting of SC and PANDAS in Aboriginal and Torres Strait Islander communities.^21^ Another explanation for this under-ascertainment of morbidity is diagnostic overshadowing when it comes to neuropsychiatric symptoms attributable to SC or PANDAS, which are quite heterogenous. Non-specific psychiatric symptoms such as irritability and inattention that occur in these post-streptococcal syndromes overlap with features of trauma-based syndromes and other neurodevelopmental disorders.^22,23^ While it is undoubtedly important to be aware of the broad range of psychiatric conditions that disproportionately affect Aboriginal and Torres Strait Islander people, it is possible that post-streptococcal neuropsychiatric sequelae may be overlooked contributors to the burden of psychiatric illness in these populations.

The paucity of epidemiological data about these conditions in Aboriginal and Torres Strait Islander people may be influenced by a range of cultural factors including culturally unsafe care that affects engagement with healthcare services and research. As such, any proposed action that seeks to define or address this under-recognised burden must be undertaken with appropriate stakeholder consultation with community members in order to promote cultural safety and to minimise harm.^24^ Empowering clinicians working in remote clinics, in particular Indigenous Health Workers, with knowledge about the potential impacts of SC and PANDAS may serve as a first step, which may then allow for further dissemination of information to increase recognition and possible diagnosis of PANDAS and SC.

Culturally safe health promotion efforts aimed at ameliorating risk factors for GAS infection, such as education about general health practices and the importance of adhering to antibiotic prophylaxis for ARF/ RHD, may also be effective in preventing the onset of SC and PANDAS. Another vital strategy to consider is training community and healthcare workers, researchers and school teachers in educational settings to be better attuned to observing and asking about neuropsychiatric manifestations of autoimmune sequelae of GAS infections in Aboriginal and Torres Strait Islander people.

## Implications for research, practice and policy

As we continue to work towards environmental health strategies, primary and secondary prevention measures targeted at eliminating GAS infections and their associated sequelae, we also need to raise awareness of post-streptococcal neuropsychiatric symptoms which may be under-reported and go undetected by children and caregivers. Further observational studies in large cohorts of Aboriginal and Torres Strait Islander people are required to better characterise the autoimmune neuropsychiatric sequelae of GAS infections in these groups. Concurrent efforts to devise standardised and culturally validated diagnostic tools are also warranted.

In addition to improving awareness, screening and detection of these conditions, the evidence base needs to be bolstered through more rigorous randomised clinical trials incorporating the known treatments for SC and PANDAS. In the case of SC, a multi-centre randomised, double-blinded, placebo control controlled trial of a 3 day course of dexamethasone entitled ‘TREAT-SC’ is about to begin across Australia and New Zealand.^25^ In Australia, where the main recruitment site will in the Northern Territory,^25^ the trial will include consultation with stakeholders in Aboriginal communities to ensure research conduct in accordance within cultural safety frameworks. While detailed information about the trial’s methodology is not yet publicly available, it is important that the trial is conducted in a manner that accounts for cultural heterogeneity across varied Aboriginal communities. From the information currently available about the trial, secondary outcomes will importantly include documentation of psychiatric symptoms using validated tools.^25^ Trials such as TREAT-SC will be important in generating evidence that will inform the management of autoimmune neuropsychiatric sequelae of GAS infections.

## Conclusion

In this piece, we have drawn attention to potentially under-investigated and under-reported sources of psychiatric morbidity attributable to GAS infections in Aboriginal and Torres Strait Islander populations. Epidemiological studies, which take into account the social, cultural and linguistic challenges contributing to the under-reporting of these conditions, could be devised to characterise and quantify this psychiatric burden. Larger scale trials aimed at investigating treatment strategies for neuropsychiatric sequelae, such as the aforementioned TREAT-SC trial, show promise in elucidating potential benefits of proposed management options. Ultimately, to best address the spectrum of autoimmune sequelae attributable to GAS infections in Aboriginal and Torres Strait Islander people, preventative public health efforts need to be fortified.
